# Metastatic Medullary Thyroid Carcinoma and Cabozantinib: Case Series and Review of Literature

**DOI:** 10.14740/wjon779w

**Published:** 2014-05-06

**Authors:** Sakshi Kapur, Han Xiao, Maureen F. Zakowski, Meera R. Hameed, Miles B. Levin

**Affiliations:** a Department of Internal Medicine, Overlook Medical Center, 99 Beauvoir Ave, Summit, NJ 07902, USA; b Division of Medical Oncology, Memorial Sloan-Kettering Cancer Center Basking Ridge, 136 Mountain View Boulevard, Basking Ridge, NJ 07920, USA; c Thoracic Pathology, Memorial Sloan-Kettering Cancer Center, 275 York Ave, New York, NY 10065, USA; d Department of Pathology, Memorial Sloan-Kettering Cancer Center, 275 York Ave, New York, NY 10065, USA; e Division of Pathology, Overlook Medical Center, 99 Beauvoir Ave, Summit, NJ 07902, USA

**Keywords:** Cabozantinib, Medullary thyroid carcinoma, Vandetanib, Calcitonin

## Abstract

Cabozantinib, a tyrosine kinase inhibitor, was approved by the US Food and Drug Administration in November 2012, for the treatment of metastatic medullary thyroid carcinoma. Although side effects typically include stomatitis, palmar-plantar erythrodysesthesia syndrome, hypertension and diarrhea, most patients are able to tolerate the recommended dose of 140 mg daily. Surgical resection is the primary treatment for medullary thyroid carcinoma. Patients with metastatic disease, who are not candidates for surgery, are considered candidates for systemic therapy. However, systemic chemotherapy has a limited role in metastatic disease. Our paper highlights not only the malignant potential of a medullary thyroid carcinoma, but also the role of cabozantinib in patients with progressive metastatic disease. We report two cases of patients with progressive metastatic medullary thyroid carcinoma (involving lung, lymph nodes, liver, pancreas, brain and spine) who responded well to therapy with cabozantinib.

## Introduction

Medullary thyroid carcinoma accounts for approximately 4% of thyroid carcinomas. Most medullary thyroid carcinomas are sporadic (75%). However, familial forms associated with multiple endocrine neoplasia type 2 (MEN2) syndrome, constitute 25% of these tumors. Systemic chemotherapy has a limited role in metastatic disease. New approaches based on molecular targeted therapies, are emerging as effective interventions for progressive disease, although most agents remain investigational. Cabozantinb, a tyrosine kinase inhibitor, has proven to be effective in patients with metastatic disease. Although most patients are able to tolerate the recommended dose of 140 mg daily, dose reductions might be needed for better patient tolerability. Currently, cabozantinib is also undergoing various clinical trials for the treatment of prostate, breast, ovary, brain, melanoma, non small cell lung, pancreatic and renal cancers.

## Case Report

### Case 1

A 40-year-old male presented in November 2012, with progressively worsening diarrhea over 6 months. Patient also noted a swelling in his right supraclavicular region for 3 months. Physical examination revealed an average sized male with no acute distress. Head and neck exam was positive for an enlarged, non tender, nodular thyroid gland, fixed to the underlying structures in the neck. Multiple lymph nodes in the head and neck region were also enlarged on palpation.

**Figure 1:**
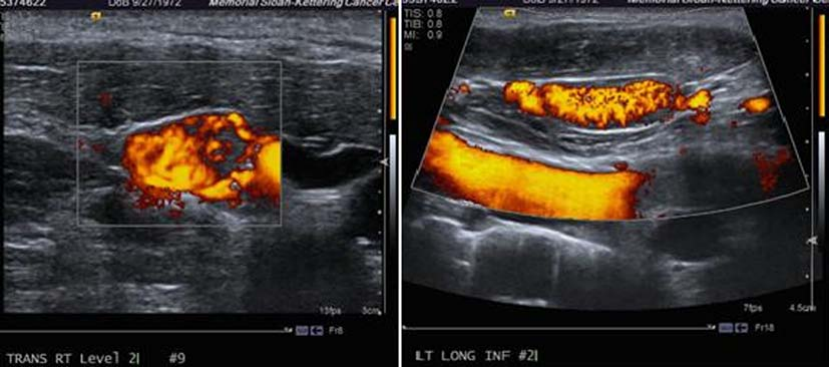
Ultrasound of the neck showing a thyroid mass suspicious of malignancy.

Ultrasound of the neck revealed: right lobe measuring 5.2 × 1.8 × 2.1 cm with a hypoechoic nodule and calcifications in the mid lobe, left lobe measuring 4.5 × 2 × 2.6 cm with a few cystic nodules and extensive bilateral cervical adenopathy (Fig. 1).

**Figure 2:**
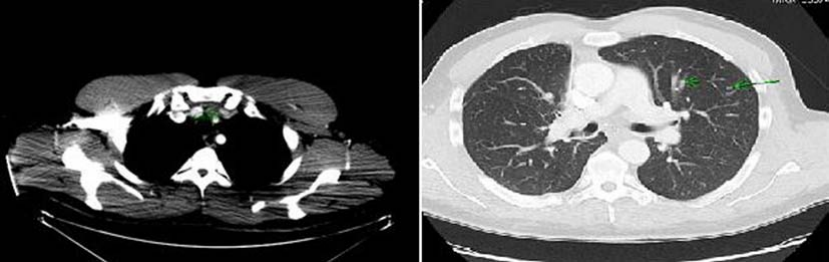
Computer tomography of the neck and chest showing a mass arising in the isthmus of thyroid gland and multiple pulmonary nodules in both lung fields.

Computer tomography (CT) of the neck and chest confirmed a heterogeneous enhancing mass measuring 2.5 × 1.3 × 3.8 cm arising from the isthmus of the thyroid gland, extensive multiple bilateral lymphadenopathy in the neck and superior mediastinum, and multiple pulmonary nodules in both lung fields (Fig. 2).

Further work-up revealed a normal complete blood count and comprehensive metabolic panel. TSH, serum calcium and parathyroid hormone level (PTH) were within normal limits. An ultrasound guided fine needle aspiration (of the thyroid gland lesion) was performed and the histopathology was consistent with a medullary thyroid carcinoma. Carcinoembryonic antigen (CEA) and calcitonin levels were 220 ng/mL (normal range: 0 - 5 ng/mL) and 19,952 pg/mL (normal range: ≤ 10 pg/mL), respectively.

**Figure 3:**
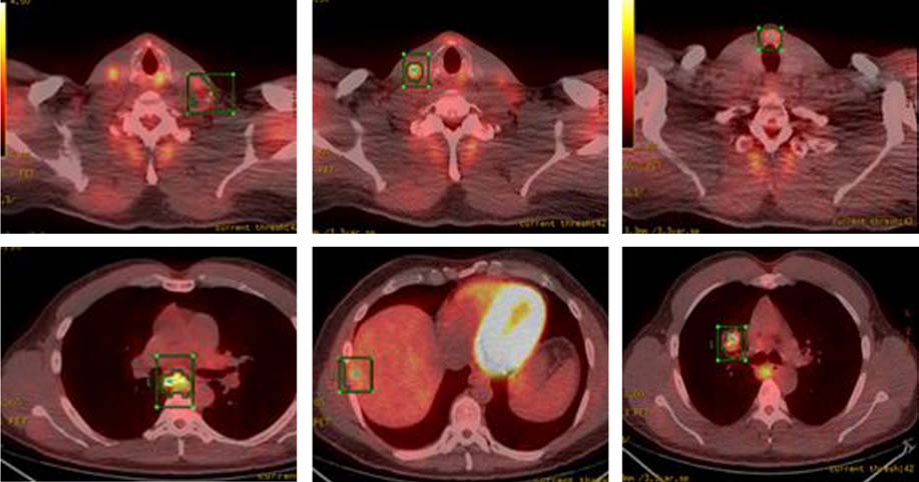
PET-CT showing FDG-avid nodular lesion in the thyroid isthmus, multiple bilateral cervical nodes, calcified mediastinal and hilar mass, patchy uptake in liver and sclerotic changes in skeleton suspicious for osseous metastasis.

Whole body PET-CT showed a calcified right suprahilar mass with SUV of 3.1. Mildly patchy FDG-avid uptake in the liver with multiple pulmonary nodules involving both lungs was seen. Sclerotic changes in the skeleton suspicious for osseous metastasis were also noted (Fig. 3). Patient was also tested for RET gene mutation and was found to be negative for the same. Serum catecholamines were within reference range.

**Figure 4:**
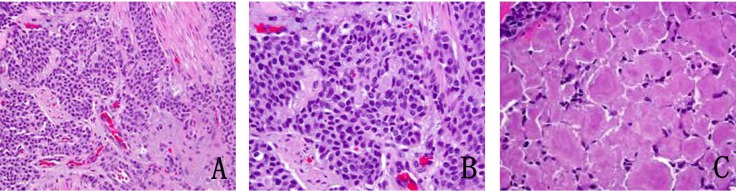
(A) Metastatic medullary carcinoma in the lung, under respiratory epithelium (× 40). (B) Higher power of metastatic medullary carcinoma in the lung (× 60). (C) Amyloid deposition in metastatic medullary carcinoma in the lung (× 60).

**Figure 5:**
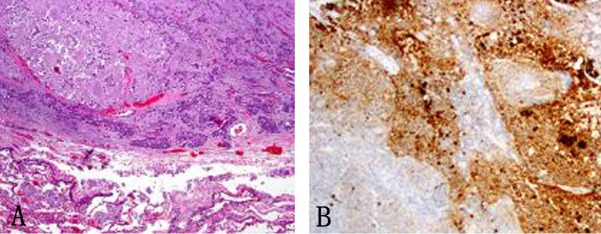
(A) Subcarinal lymph nodes positive for metastatic disease (× 40). (B) Synaptophysin stain on lymph node metastatis (× 40).

In January 2013, patient underwent a total thyroidectomy with bilateral modified neck dissection as a palliative surgical procedure. Although he did well after surgery, his serum calcitonin (> 500 pg/mL) and CEA levels (> 150 ng/mL) continued to remain high. In February 2013, a right upper lobectomy with mediastinal lymph node dissection was performed for a large suprahilar calcified mass in the right upper lobe of the lung. Surgical pathology confirmed metastatic medullary thyroid carcinoma with amyloid deposition. Although the margins were negative, both lymphatic invasion and pleural involvement were noted. Sub carinal (right level 10 and right level 4) lymph nodes were also positive for metastatic disease (Fig. 4, 5).

**Figure 6:**
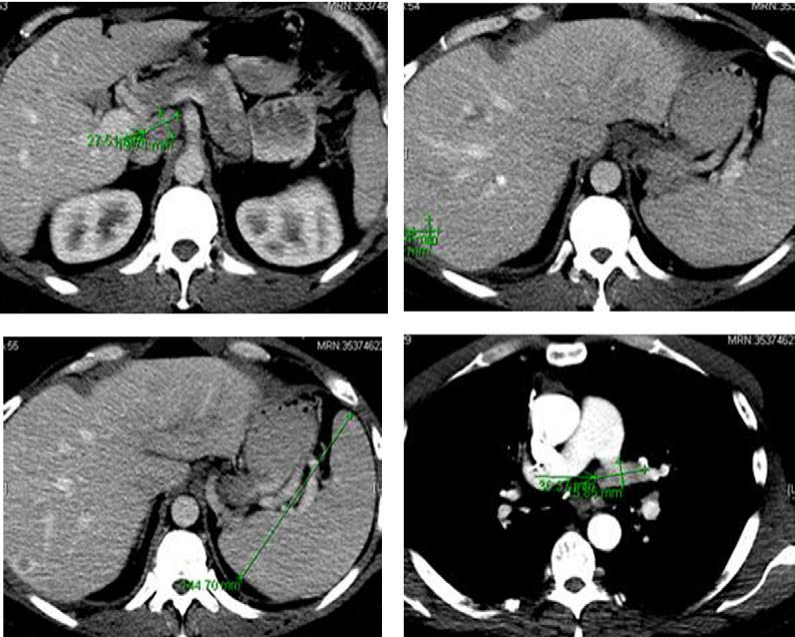
Computer tomography of the abdomen showing several hepatic metastasis, splenomegaly and abdominal adenopathy.

Following the surgery, in March 2013, patient developed multiple episodes of pancreatitis (waxing and waning). Endoscopic ultrasound revealed multiple metastatic lesions in the pancreas (3 mm in head, 2 mm and 3 mm in neck and 1 mm in tail) and the liver. Multiple enlarged lymph nodes, especially at the level of porta hepatis, were also noted (Fig. 6). Fine needle aspiration of these lesions confirmed malignant cells consistent with medullary thyroid carcinoma. Gastroenterology was consulted and two pancreatic stents were inserted.

**Figure 7:**
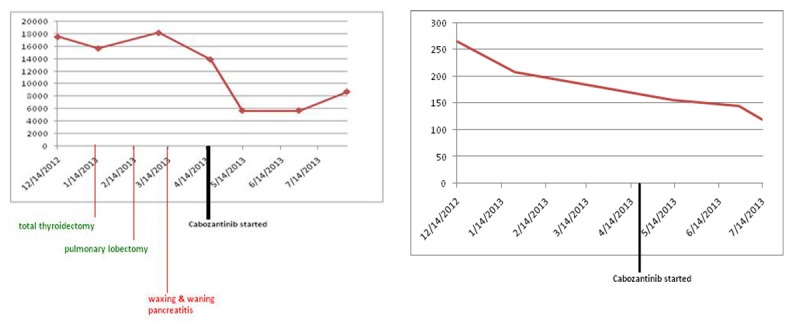
Both serum calcitonin and CEA levels following cabozantinib therapy. Y axis: serum calcitonin levels pg/mL; Y axis: serum CEA levels ng/mL.

Since patient’s disease continued to progress, we decided to start the patient on cabozantinib. In April 2013, patient was started on 140 mg of cabozantinib daily. Nine months later, he continues to follow up as an outpatient and has been tolerating cabozantinib well. Both serum calcitonin and CEA levels have trended down (Fig. 7). Repeat imaging also shows stabilization of his disease. We continue to monitor the patient by checking tumor markers (serum calcitonin and CEA), and repeat imaging (CTs) every 3 months to look for any progression of his disease.

### Case 2

A 62-year-old female presented to our hospital with progressively worsening lethargy and neck swelling for 3 months. Head and neck exam was positive for thyromegaly and multiple bilateral cervical lymphadenopathy. Fine needle aspiration revealed results compatible with medullary thyroid carcinoma.

Further work-up showed a normal complete blood count and comprehensive metabolic panel. Serum calcium, parathyroid hormone level, TSH and serum catecholamines were within reference range. Serum calcitonin and CEA levels were 1,619 pg/mL (< 5.0 pg/mL) and 435 ng/mL (0 - 5 ng/mL), respectively. She also tested negative for RET mutation.

**Figure 8:**
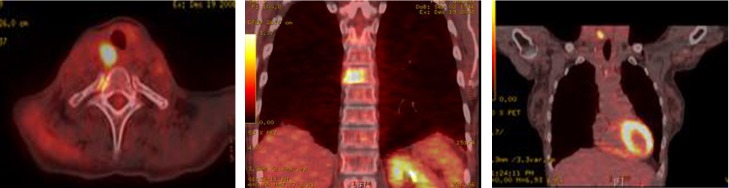
PET-CT showing extensive metastatic disease involving the spine.

A total thyroidectomy with bilateral lymph node dissection along with superior mediastinal lymph node dissection was performed. Histopathology results confirmed medullary thyroid carcinoma. However, 3 months later, PET-CT showed right tracheoesophageal groove recurrence with diffuse spinal metastasis (Fig. 8).

**Figure 9:**
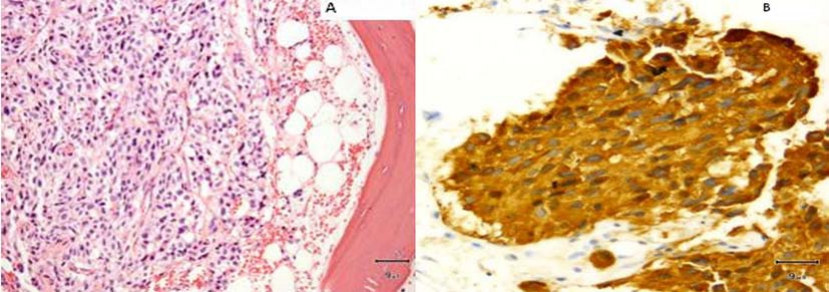
(A) Vertebral biopsy showing metastatic medullary thyroid carcinoma. (B) Synaptophysin stain on vertebral metastasis.

A core biopsy of the T8 vertebral body confirmed metastatic medullary carcinoma involving the spine (Fig. 9). Both zoledronic acid and denosumab were started. Patient received radiation to her T8-T9 vertebrae. Although patient did well, her serum calcitonin levels (> 500 pg/mL) continued to remain elevated.

**Figure 10:**
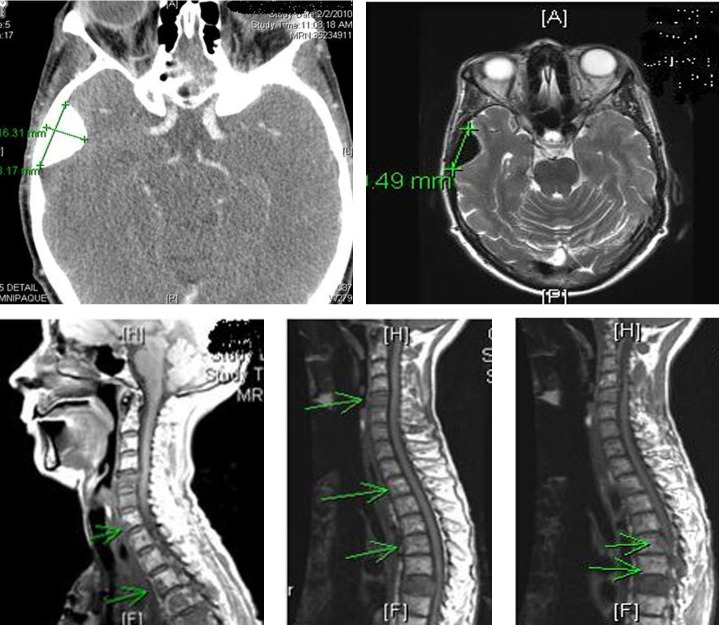
Computer tomography of the head and spine region showing left orbital metastasis and worsening spinal metastasis.

Patient received multiple cycles of chemotherapy (dacarabzine 60 mg/m^2^ and 5-FU 400 mg/m^2^), over the next 18 months. However, because of worsening lethargy, patient’s chemotherapy was stopped. Repeat computer tomography of the head and spine showed worsening spinal metastasis and metastasis to the left orbit (Fig. 10).

**Figure 11:**
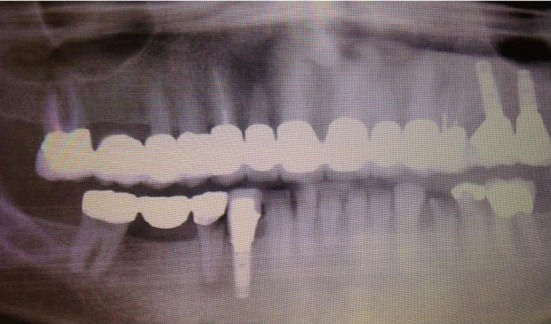
Osteonecrosis of the jaw (ONJ) secondary to bisphosphonates.

She received radiation to her left orbit, L5 sacrum and T2-T5 vertebrae. Both zoledronic acid and denosumab were continued. However, patient developed osteonecrosis of her jaw (ONJ) secondary to bisphosphonates (Fig. 11). A dental consultation was obtained and both zoledronic acid and denosumab were stopped.

**Figure 12:**
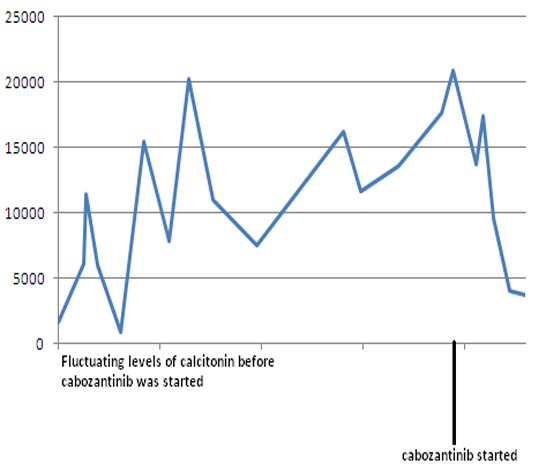
Serum calcitonin levels following cabozantinib therapy. Y-axis: serum calcitonin in pg/mL.

Because of worsening metastatic disease, patient was started on cabozantinib (140 mg daily). However, she developed worsening diarrhea, so cabozantinib was held. She was restarted on a lower dose of 20 mg daily and was gradually increased to 60 mg daily. Eight months later, the patient continues to follow up as an outpatient and her disease has been stable so far. Both serum calcitonin and CEA levels have trended down following cabozantinib therapy (Fig. 12). Repeat imaging also shows stabilization with no progression in her disease.

## Discussion

Medullary thyroid cancers (MTCs) are neuroendocrine tumors arising from parafollicular cells (C cells) in the thyroid gland. They can either occur sporadically (75%) or as a part of multiple endocrine neoplasia (MEN) type 2 syndrome (25%). MTC can be classified as follows: A solitary thyroid nodule is the most common presentation of sporadic MTC (75-90%) (Table 1) [1, 2]. Approximately 50% of the patients have nodal enlargement, up to 15% have symptoms of upper aero digestive compression, such as dysphagia and hoarseness, and about 5% have distant metastatic disease [3]. Most common sites for metastasis include the liver, lung, bones, and less often, brain and skin. In most patients with MTC, the disease is already metastasized at the time of diagnosis.

MTCs secrete both calcitonin and CEA, both of which can serve as tumor markers. However, Barbet et al in a study reported calcitonin doubling time as a better predictor of MTC survival than CEA [4]. Systemic symptoms can occur due to hormonal secretion by the tumor. Calcitonin and calcitonin-gene related peptide can cause diarrhea or facial flushing in patients with advanced disease. Rarely ectopic ACTH secretion can cause Cushing’s syndrome in patients with MTC.

In general, patients with MTC are treated primary with surgical resection. Complete resection of the thyroid gland and any local and regional metastasis is required to cure these patients. Serum calcitonin and CEA levels should be measured 2 - 6 months after surgery to detect the presence of residual disease. In a large series by Lee et al, patients with undetectable calcitonin values following surgery, had 5-year recurrence rate of only 5% [5]. Up to 50% of the patients have persistent hypercalcitoninemia following surgery [6, 7]. Calcitonin values < 150 pg/mL, usually indicate persistent loco-regional disease in the neck. However, values >150 pg/mL following surgery suggest distant metastasis [1].

Patients with progressive or symptomatic metastatic disease, who are not candidates for surgery, are considered candidates for systemic therapy. Traditional cytotoxic agents provide limited benefit. Most regimens for patients with MTC combine dacarbazine with other agents, such as vincristine, 5-FU, cyclophosphamide, streptozocin or doxorubicin without significant advantage of one combination compared with another [8]. New approaches based on molecular targeted therapies, are emerging as effective interventions for progressive disease, although most agents remain investigational.

Cabozantinib (XL 184) is an oral, small molecule tyrosine kinase inhibitor that targets VEGF receptors 1 and 2, c-MET and RET [9]. It was approved by the US Food and Drug Administration, in November 2012 for the treatment of metastatic MTC [10]. In a phase I dose escalation study, 10 of the 35 patients (29%) achieved a confirmed partial response. Stable disease of at least 6-month duration was seen in 40% of the patients. The overall rate of partial response and 6-month progression free survival was 68%. Responses were noted in patients regardless of the RET mutation status [11]. In another trial, 330 patients with metastatic MTC were randomly assigned to either cabozantinib (140 mg) or placebo group once daily. A significant prolongation in progression free survival was observed in the cabozantinib group [12]. The recommended dose of cabozantinib is 140 mg daily (with dose reductions to adjust for tolerability). Side effects typically include: stomatitis, palmar-plantar erthyrodysesthesia syndrome, hypertension and diarrhea. Rarely, patients can develop fistula formation and ONJ. Our second patient developed ONJ before staring cabozantinib therapy, secondary mainly to bisphosphonates.

Cabozantinib in a phase II trial in 2009, showed encouraging results with relapsed glioblastoma multiforme. Cabozantinib has also shown to be beneficial in metastatic advanced castration resistant prostate cancer. It is currently undergoing various clinical trials for the treatment of prostate, breast, ovary, brain, melanoma, non small cell lung, pancreatic and renal cancers.

Vandetanib (ZD6474) is an oral inhibitor that targets VEGF receptors, RET and EGF receptors. It is also approved by the US Food and Drug Administration, for the treatment of metastatic MTC. Recommended dose is 300 mg daily and side effects typically include: diarrhea, colitis, rash, hypertension and very rarely prolonged QTc interval/torsades de pointes. In a randomized phase III trial of vandetanib (300 mg daily), patients with CEA doubling times greater than 24 months, were unlikely to benefit from this drug. Also, patients who were positive for an RET M918T mutation had an improved progression free survival [13].

### Future trends

Motesanib, is a highly selective tyrosine kinase inhibitor that targets all three VEGF receptors, platelet derived growth factor receptor and RET. In a phase II study, 91 patients with advanced MTC were treated with motesanib (125 mg daily) for up to 48 weeks. Although partial response was noted in only two patients out of 91, 48% of the patients had stable disease for at least 24 weeks [14]. However, more research is needed before motesanib can be widely accepted for the treatment of metastatic MTC.

Radioimmunotherapy is another field that needs to be explored for treating MTC. In a non randomized trial, patients with progressive metastatic MTC were given anti-CEA/anti-diethylenetriamine pentaacetic acid recombinant bispecific antibodies. The median overall survival after administration of this therapy was 110 months, compared to 60 months in the untreated cohort [15].

### Conclusion

MTC can have a very malignant course with involvement of various organs, such as the lymph nodes, liver, lungs, bones and rarely brain. Approximately 50% of the patients have metastatic disease on diagnosis. Cabozantinib, a tyrosine kinase inhibitor, has been found to be an effective treatment option for patients with metastatic MTC. Both our patients had stabilization of their disease on cabozantinib. Although the optimum recommended dose is 140 mg daily, dose reductions might be needed for better tolerability in some patients.
